# Mindfulness training modulates EEG oscillations and improves shooting accuracy in competitive stress

**DOI:** 10.3389/fpsyg.2026.1802645

**Published:** 2026-05-18

**Authors:** Weitao Li, Jiawen Guo, Meiting Wei, Guangheng Dong

**Affiliations:** 1 School of Physical Education, Yunnan University, Kunming, Yunnan, China; 2 Chinese Language and Culture College, Huaqiao University, Xiamen, Fujian, China; 3 School of Psychology, Central China Normal University, Wuhan, Hubei, China; 4 Center for Educational Cognitive Neuroscience, Faculty of Education, Yunnan Normal University, Kunming, Yunnan, China

**Keywords:** athletic performance, attention, competitive stress, mindfulness training, shooting

## Abstract

**Background:**

Rifle shooting requires low arousal and precise motor control. Mindfulness training is theorized to support these demands, but systematic evidence of its neurocognitive and performance benefits under authentic competitive stress in elite shooters is scarce. This study aimed to examine whether a 7-week Mindfulness-Acceptance-Insight-Commitment (MAIC) program enhances attentional neural efficiency and shooting performance in elite 10m air rifle athletes under standardized competitive stress.

**Methods:**

Fourteen elite shooters were randomly allocated to a mindfulness group (*n* = *7*) or a control group (*n* = 7). The mindfulness group completed two MAIC sessions alongside routine training per week for 7 weeks. All participants underwent testing at baseline (A1), post-intervention (A2), and 2-week follow-up (A3) in an ISSF-compliant range under a validated stress-induction protocol. Measures included shooting scores (SIUS LS10), state anxiety (CSAI-2), dispositional mindfulness (FFMQ), and 64-channel EEG spectral power (δ, θ, α, β, γ bands). Behavioral data were analyzed using repeated-measures ANOVA and Spearman correlation. EEG data were analyzed using Aligned Rank Transform ANOVA to accommodate non-normal data distributions in factorial designs.

**Results:**

Stress induction successfully elevated anxiety in both groups (*p* < 0.001). The mindfulness group exhibited a significantly greater increase in mindfulness scores compared to the control group. For EEG measures, a significant group × time interaction was observed for α-band power, *F*_(2, 24)_ = 5.62*, p* = 0.028, indicating enhanced top-down attentional control in the mindfulness group. For δ*-*band power, a significant main effect of time was found, *F*_(2, 24)_ = 5.35, *p* = 0.012, with both groups showing reductions over time, reflecting a shared pattern of task habituation and optimized neural efficiency. For shooting performance, despite lacking a significant interaction, the mindfulness group showed a descriptive, non-significant within-group improvement across time points, and scored significantly higher than the control group at post-test.

**Conclusions:**

A 7-week MAIC intervention may be associated with improvements in attentional regulation, specific changes in EEG oscillatory activity (increased α), and a descriptive within-group enhancement in shooting performance under competitive stress in elite rifle athletes. The programme offers an evidence-based, sustainable psychological training model for precision sports.

## Introduction

Maintaining attentional focus under competitive stress is a critical challenge in precision sports like rifle shooting. In this neurocognitive context, attentional focus is not merely generalized attention, but the goal-directed cognitive ability to sustain neural resources on task-relevant cues (Corbetta and Shulman, 2002) while actively resisting internal and external distractions (Bonnefond and Jensen, 2025). Under such high-stakes demands, empirical evidence confirms that elite shooters must maintain sustained attention (Pei et al., 2022)—defined in cognitive neuroscience as the capacity to continuously allocate cognitive resources toward goal-directed actions over time, a process fundamentally supported by rhythmic neural oscillations modulating cortical excitability (Buschman and Kastner, 2015; Helfrich et al., 2018)—while controlling fine motor responses in low-arousal states (Chen et al., 2022; Yu et al., 2024). However, performance pressure often elevates arousal beyond the optimal “inverted-U” zone (Zhao and Zhang, 2025), disrupting the “cortical quieting” central to the psychomotor efficiency hypothesis (Hatfield, 2018), where even minimal attentional lapses may lead to performance failure (Beilock and Carr, 2001). In such contexts, conventional psychological skills training (e.g., self-talk, relaxation) may have limitations, as it tends to emphasize the control or suppression of negative internal experiences rather than accept them (Gardner and Moore, 2004).

Mindfulness training has emerged as a promising alternative. Importantly, rather than being a single skill, mindfulness is conceptualized as a multi-component construct that typically integrates attentional focus, emotion regulation, and meta-awareness. Defined as “paying attention in a particular way: on purpose, in the present moment, and non-judgmentally” (Kabat-Zinn, 1994), mindfulness fosters meta-awareness and acceptance of internal experiences. In sports, it has been shown to improve attentional control, emotional regulation, and resilience under pressure (Reinebo et al., 2024). This is particularly vital in closed-skill sports, where “mild acceptance” allows athletes to observe anxiety without the reactivity that disturbs fine motor control (Meeûs et al., 2010). Evidence from mindfulness-based programmes—such as mindfulness acceptance commitment (MAC) and mindful sport performance enhancement (MSPE)—has shown improvements in attentional stability and competitive outcomes across various sports (Bühlmayer et al., 2017). However, whether mindfulness can effectively support attentional stability in precision sports characterized by low arousal (Jekauc et al., 2021) and extreme attentional demands remains insufficiently understood.

From a neurocognitive perspective, sustained and goal-directed attention in precision sports such as rifle shooting primarily relies on coordinated activity across frontal and parietal cortical regions (Cheng et al., 2023, 2026). Rather than being static, this network mediates the rhythmic spatial attention necessary for sustaining focus over time (Fiebelkorn et al., 2018), playing a central role in top–down attentional control and the maintenance of task goals. Specifically, the prefrontal cortex is mainly involved in executive control and attentional regulation, while parietal regions contribute to attentional allocation and the integration of sensory information during visually guided actions (Corbetta and Shulman, 2002). Neurophysiological evidence further indicates that oscillatory activity in frontal and parietal regions—particularly within the alpha (8–12 Hz) and delta (1–4 Hz) frequency bands—is closely associated with attentional stability. Alpha power functions as an active gating mechanism to sharpen visual awareness (Yang, 2025), and is associated with internally directed attention, whereas delta activity reflects fundamental homeostatic and motivational drives (Knyazev, 2012), where increased power may signal attentional disengagement or cognitive load (Klimesch, 2012). Accordingly, examining EEG oscillatory activity in frontal and parietal regions provides a theoretically and empirically grounded approach for investigating how mindfulness training modulates attentional processes during rifle shooting.

While accumulating evidence from neuroimaging studies—such as the fNIRS work by Gao and Zhang (2023) linking brief mindfulness to prefrontal efficiency in novice shooters—highlights its cognitive benefits, most existing findings are derived from laboratory-based settings or low-pressure tasks (Gao and Zhang, 2023). Moreover, although recent virtual reality protocols have helped isolate psychomotor determinants of shooting (Buga et al., 2025), and high-fidelity datasets now underscore the necessity of multimodal monitoring to capture the complexity of elite concentration (Pei et al., 2022), it remains unclear whether these effects generalize to the “cool and focused” neural state required in elite shooting (Milton et al., 2007). Consequently, a substantial gap remains in our understanding of the neurocognitive mechanisms through which mindfulness operates under ecologically valid, competition-like conditions.

In light of this background, the present study selected the 10 m Air Rifle event as an ideal model for examining the attentional benefits of mindfulness training. This discipline places exceptionally high demands on sustained, goal-directed attention while involving minimal motor variability, thereby allowing for precise assessment of intervention effects. The current study aims to investigate how a 7-week mindfulness training programme, based on the Mindfulness Acceptance Insight Commitment (MAIC) framework, influences attentional regulation and shooting performance in elite rifle athletes under simulated competitive stress. By integrating behavioral performance measures, subjective assessments, and EEG indices of attention, this study seeks to elucidate the neural and psychological mechanisms through which mindfulness enhances performance in high-stakes precision sports. Based on these theoretical considerations, we formulated two specific, directional hypotheses. First, we hypothesized that the mindfulness training group would demonstrate an improvement in dispositional mindfulness and shooting performance under competitive stress compared to the control group. Second, at the neurocognitive level, we hypothesized that mindfulness training would enhance top-down attentional regulation and neural efficiency. Specifically, we expected this to be reflected by an increase in attention-related neural indices.

## Methods

### Ethics

The study was approved by the Ethics Committee of Yunnan University (Approval No. YNUHM2025003), and written informed consent was obtained from all participants in accordance with the Declaration of Helsinki.

### Design

The study employed a 2 (Group: mindfulness vs. control) × 3 (Time: pre-test, post-test, follow-up) mixed factorial design. A single-blind (outcome assessor) design was implemented to minimize expectancy and observer bias. While participants could not be blinded to the intervention condition due to the nature of the training, they were naive to the specific research hypotheses and were only informed that the study examined psychological factors related to shooting performance. Referees responsible for data collection were blinded to group allocation and adhered strictly to standardized testing procedures. They provided no technical feedback or psychological guidance at any stage of the study, ensuring that all outcome measures were obtained objectively and without contamination from experimenter influence. Randomization was computer-generated, with participants randomly assigned to one of the groups.

### Participants

A total of 20 elite rifle shooters were initially recruited from a provincial shooting and archery training center in China. Following preliminary screening, six athletes were excluded due to injury, national team obligations, or preparation for international competitions. The final sample consisted of 14 athletes (9 males, 5 females; mean age = 20.25 ± 2.24 years), all of whom were provincial champions holding at least National First-Level certification (including 11 National Master-Level athletes and 3 National First-Level athletes). All athletes were healthy, right-handed, and free from injuries or neurological conditions that could affect shooting performance or EEG recordings. None had received prior mindfulness or psychological skills training within the past 6 months.

### Behavioral measurements

The Competitive Sport Anxiety Inventory-2 (CSAI-2) was used to assess athletes' competitive anxiety. The scale measures three components—cognitive anxiety, somatic anxiety, and self-confidence—and is widely applied in sport psychology research due to its strong reliability and validity. Its sensitivity to pre-competition psychological fluctuations makes it a standard tool for evaluating anxiety-related mechanisms and intervention effects (Martinent et al., 2010).

The Five Facet Mindfulness Questionnaire (FFMQ) was employed to measure participants' dispositional mindfulness. The FFMQ assesses five facets—observing, describing, acting with awareness, non-judging, and non-reactivity—and has been extensively validated across mindfulness research. Its multidimensional structure allows for a comprehensive evaluation of trait mindfulness (Choi, 2015).

Shooting performance was assessed using the LS10 electronic scoring system (SIUS, Switzerland), which recorded the score and displacement of each shot with a precision of 0.1 rings. In accordance with ISSF regulations, participants completed a series of 60 shots with a standard 10 m air rifle, yielding a maximum possible score of 654.0 rings. The system provided real-time tracking of shot scores and intervals, with data automatically uploaded to a secure online database for storage. Throughout testing, a certified referee monitored the procedure to ensure adherence to official competition rules; no technical guidance or psychological intervention was provided by the referee during the session.

### Procedure

The study was conducted over a 14-week period. The procedure consisted of four phases: a pre-test (A1), a 7-week mindfulness intervention phase (B), a post-test (A2), and a follow-up assessment (A3). All experimental sessions took place at the shooting training center. Measurements were carried out in a 10 m air rifle range that complied with international shooting sport federation (ISSF) competition standards. Environmental conditions were strictly controlled and maintained as follows: shooting lane illumination at 800–1200 lux, ambient illumination at 300–500 lux, temperature at 22 ± 2 °C, and relative humidity at 50% ± 10%. Prior to each testing session, participants completed a 10-min warm-up followed by 15 min of trial shooting to acclimatize to the range conditions.

**Figure 1:**
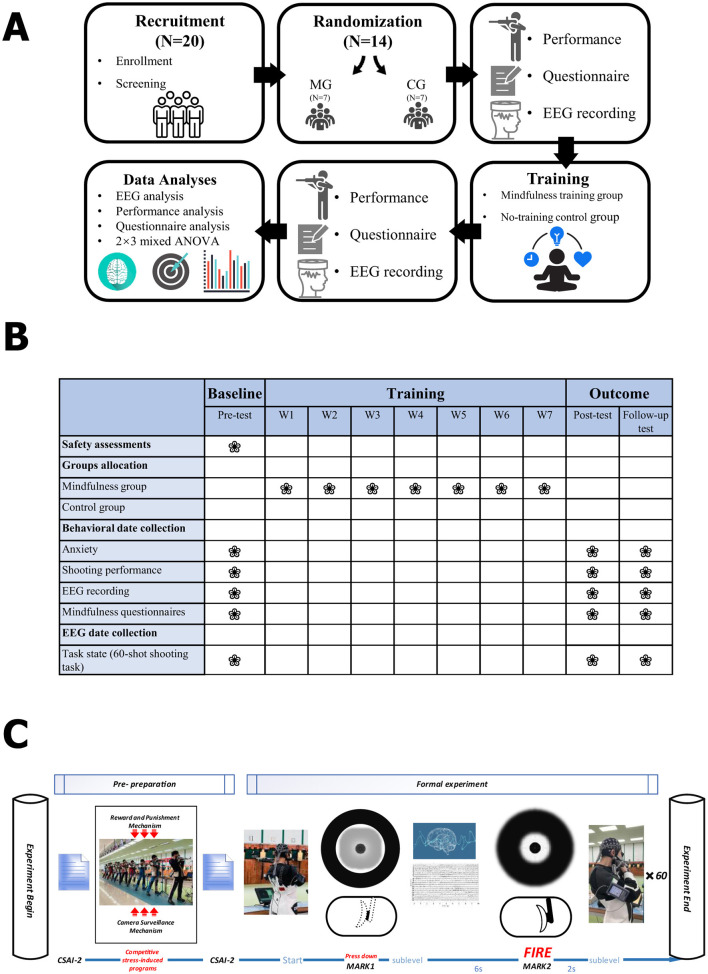
Overview of the study design and experimental procedure. **(A, B)** Flowchart of the study design and measurement timeline. This figure illustrates the overall structure and time schedule of the study. A total of *N* = 20 elite rifle athletes were initially recruited; following screening, the final sample of *N* = 14 participants was computer-randomized to either the mindfulness training group (MG, *n* = 7) or the control group (CG, *n* = 7). All participants underwent pre-test measurements, including performance assessment, Questionnaire completion, and EEG recording. The training phase lasted 7-weeks, during which the MG received the MAIC-based mindfulness intervention, and the CG continued with routine training. A post-test was conducted immediately after the intervention, followed by a Follow-up test 2 weeks later. Final data analyses employed a 2 × 3 mixed factorial design ANOVA, covering shooting performance, questionnaire scores, and EEG indices. **(C)** Trial flowchart for the competitive stress-induced task and EEG marker description. This figure details the formal experimental procedure conducted under a competitive stress-induced environment. The experiment began with a pre-preparation phase that introduced a competitive stress-induction procedure, including reward and punishment mechanisms and camera surveillance mechanisms. The CSAI-2 questionnaire was collected before the stress induction, and both the CSAI-2 and FFMQ were administered post-induction to verify the stress manipulation effectiveness and assess mindfulness levels under pressure, respectively. In the Formal experiment, participants were required to complete 60 scored shots (times 60) in a standing position. Each trial followed a sequence that generated specific electrophysiological markers: Start → Entering Aiming Phase (Marker MARK1) → 6 s delay → Formal Shot (Marker MARK2) → 2 s delay. EEG Data Segmentation: Both MARK1 (trigger pull entering the aiming phase) and MARK2 (instant of formal shot) served as EEG event markers. EEG data were segmented using MARK2 (the instant of the shot) as the time zero, saving 6 s backward and 2 s forward for analysis.

The study flowchart is shown in Figure 1.

### The mindfulness comprises four sequential phases

#### Pre-test (A1)

This study employed the 10 m air rifle standardized competition protocol certified by the ISSF. A structured pressure-inducing environment was created through a combination of reward–punishment mechanisms and social observation to simulate competitive stress. Under this condition, baseline psychological (mindfulness level and stress level), physiological (EEG signals), and performance (shooting scores) data were collected from the athletes.

Before the formal shooting task began, athletes entered a quiet testing room to complete the CSAI-2 independently to assess their baseline competitive anxiety. After the initial assessment, the coach presented the anxiety-induction procedure to elicit competitive stress. Athletes then completed the CSAI-2 and the FFMQ again to verify whether the induction successfully increased anxiety and to evaluate their mindfulness level under stress. The entire questionnaire process took approximately 10–15 min.

During the preparation phase, each athlete used either a Feinwerkbau 800 series (manufactured in Germany). These rifles meet the standard 4.5-mm caliber specifications. The ammunition used was QIANG YUAN pellets. Targets followed the standard concentric ring format, with ISSF-standard 10 m air rifle targets. Under the supervision of a referee, athletes first completed a 15-min warm-up shooting session to allow acclimatization to the equipment and ensure rifle stability under the environmental conditions.

During the formal test, each athlete was required to perform 60 scored shots in a standing position following ISSF standards. There was no restriction on the number of adjustments allowed during shooting. All recorded scores contributed to the final result. The total shooting duration, including warm-up and scoring, was capped at 75 min. Timekeeping was managed by the HS-70W-8 electronic timing system (CASIO, Japan) to ensure compliance with international regulations. Shooting performance was captured by the LS10 electronic scoring system (SIUS, Switzerland), which recorded scores to one decimal place (e.g., 10.9) and displayed results in real time on an observation screen.

To enhance ecological validity, this study adopted an empirically validated pressure manipulation protocol based on existing literature (Peng, 2021). A realistic high-pressure competitive environment was simulated during the test phase. Before shooting began, participants were informed that the session would follow official match procedures and that results would be ranked and published. Throughout the test, athletes' scores were continuously updated and publicly displayed, with each athlete being informed of their current rank. This approach served to heighten the sense of competition. In addition to scoring via the LS10 system, technical performance was recorded using an FDR-AX45A video camera positioned 2 m in front of the athlete. The footage was used for subsequent technical analysis by coaches. To further simulate a pressure-filled competitive atmosphere, spectators—including coaches and administrative personnel—observed the test live. Anxiety levels were monitored using the CSAI-2 to verify the effectiveness of the pressure manipulation. To intensify competitive pressure, both reward and punishment mechanisms were introduced. Cash prizes of ¥100, ¥80, and ¥50 were awarded to the top three performers, respectively. Athletes ranked near the bottom received negative feedback on their performance. After each scoring series (10 shots), experimenters provided athletes with real-time ranking updates and verbal prompts such as: “The scores are very close—please focus and try to improve your performance in the next round, or you may face elimination.” Athletes who ranked last after the full 60-shot session were required to complete a 5,000-m run as a penalty (this penalty was used as a verbal threat only and was not actually implemented), thereby further increasing performance anxiety and stress levels.

#### Intervention (B)

All athletes maintained regular shooting training (five sessions per week) with matched content and intensity. The mindfulness group additionally completed a structured mindfulness intervention, whereas the control group continued routine training only. Details of the intervention are provided in section Mindfulness interventions.

#### Post-test (A2)

The post-test followed the identical procedures, timing, and measurement tools used in the pre-test (A1) to ensure comparability. Testing occurred immediately after the 7-week intervention period.

#### Follow-up test (A3)

A follow-up test was conducted 2 weeks after the intervention to assess sustained effects. Procedures were identical to A1 and A2. During this period, the mindfulness group returned to routine training without additional intervention.

### Interventions

#### Mindfulness group

Athletes in the mindfulness group received a 7-week structured mindfulness programme based on the MAIC framework. The programme consisted of 14 sessions (two per week, ~50 min each). Training incorporated core mindfulness practices—including breath awareness, body scanning, and focused attention—integrated with sport psychology principles to enhance attentional control, cognitive regulation, and emotional stability under competitive pressure.

All sessions were exclusively delivered by certified sport psychology professionals with formal mindfulness training and extensive experience working with shooting athletes. The protocol followed standardized procedures to ensure methodological consistency and intervention fidelity. To ensure strict control over the volume of mindfulness practice and prevent unmonitored extracurricular training, the proprietary audio guides and training materials used during the sessions were retained by the research team and not distributed to the athletes. During the baseline screening phase, it was inferred from observations that the high-intensity training regimens and strict daily schedules of these elite athletes would likely preclude the feasibility of unstructured, self-directed practice. Furthermore, to avoid the “ironic rebound effect” (i.e., paradoxically increasing attention to a behavior by explicitly forbidding it), participants were not formally prohibited from engaging in private practice. Instead, they were informed that technical support would be provided upon request should they wish to practice outside of formal sessions. Notably, no such requests were made by any participant throughout the 7-week intervention period. The full intervention schedule is provided in Table 1.

**Table 1:** MAIC mindfulness training intervention plan.

Session module	Topic	Content	Practice content
1	Mindfulness training preparation	Introducing the content of the mindfulness course basic exercises and tips, and mindfulness level test	Mindfulness concentration exercises
2	Mindfulness	The concept of mindfulness and the core factors of mindfulness	Mindfulness and breathing practice
3	Mindful attention and awareness	Mindful attention: mindful awareness	Mindful breathing, body scan and mindfulness meditation practice
4	Mindful acceptance	Introduction to mindful acceptance: explaining experience acceptance and avoidance	Coexistence skills exercises, decision skills exercises, and mindfulness yoga exercises
5	Values and consciousness	Introduce the relationship between values and goals	Practice mindful breathing and meditation during shooting
6	Investment	What is investment and concentration?	Practice mindfulness meditation and mindfulness training in stressful situations
7	Comprehensive	Review and synthesis of mindfulness training, emphasizing the continuity of mindfulness training and its fit with oneself	

#### Control group

Athletes in the control group received no psychological intervention and continued with their regular training routines, serving as an active training control to minimize potential confounding effects.

### EEG recording and preprocessing

EEG data were recorded during the formal testing phase using the NeuSen W wireless EEG headset (Brain-Computer Technology Co., Ltd, Neuracle, Changzhou, China), a 64-channel dry-electrode system positioned according to the extended international 10–20 system. All channels were referenced to a point between the Fz and FCz electrodes, and offline re-referencing was conducted to the average of all electrodes (average reference). The ground electrode was placed on the forehead anterior to the Fz position. The device operates with dry electrodes that maintain acceptable impedance levels. The sampling rate was set at 1,000 Hz. During data acquisition, scalp impedance for each electrode was maintained below 5 kΩ. The bandpass filter was configured between 0.01 Hz and 100 Hz.

All EEG data were processed using MATLAB 2023b (MathWorks) and the EEGLAB toolbox (version 2024.0) (Delorme and Makeig, 2004). Initial preprocessing involved filtering the data using EEGLAB's “Basic FIR filter” function. A bandpass filter was applied with a lower cut-off frequency of 0.1 Hz and an upper cut-off of 40 Hz. Additionally, a notch filter was applied at 49–51 Hz to remove power line interference. The data were then re-referenced to the average reference. Independent component analysis (ICA) was used to decompose the EEG signals into components representing muscle activity, ocular artifacts, cardiac activity, line noise, and channel noise (Delorme et al., 2007). Components classified as non-neural (e.g., muscle, eye, heart, or noise) were removed using EEGLAB's “Remove components from data” function, leaving only “brain” and “other” components. The “other” components were retained as they may include residual brain-related activity. Consequently, exactly 60 trials (shots) were retained for the final analysis for each participant across all testing phases.

### Frequency analysis of EEG data

EEG data were further analyzed in the frequency domain to examine training-related changes in neural oscillatory activity. Following artifact correction and preprocessing, power spectral density (PSD) was computed using Fast Fourier Transform (FFT) implemented in EEGLAB. Spectral power was calculated for each electrode and then averaged within predefined frequency bands: delta (1–4 Hz), theta (4–8 Hz), alpha (8–12 Hz), beta (12–30 Hz), and gamma (30–40 Hz).

To reduce inter-individual variability and enhance functional interpretability, electrodes were grouped into region-of-interest (ROI) clusters based on their anatomical locations. For this purpose, the channels (F3, Fpz, F4) over the frontal region, the channels (C3, Cz, C4) over the central region, the channels (P3, Pz, P4) over the parietal region, the channels (T7, T8) over the temporal region, and the channels (O1, Oz, O2) over the occipital region were grouped into respective clusters. These five cortical regions were defined and included in the global omnibus ANOVA: frontal, central, parietal, temporal, and occipital regions. However, while the overarching statistical model included all five regions to capture global spatial dynamics, our specific *a priori* hypotheses and subsequent correlational analyses focused exclusively on the frontal and parietal networks. While shooting tasks naturally recruit widespread cortical networks including visual and motor areas, these two specific ROIs were selected to directly target the top-down attentional control mechanisms hypothesized to be modulated by mindfulness training (Scolari et al., 2015). Mean spectral power within each frequency band was averaged across electrodes belonging to the same ROI for each participant and each testing phase.

### Statistical analysis

All statistical analyses were conducted using R (version 4.5.2; R Core Team) and RStudio. Assumption testing indicated that the homogeneity of variance was satisfied (Levene's test). Normality was assessed using the Shapiro–Wilk test, which revealed that EEG data violated the normality assumption, whereas shooting performance and mindfulness variables were normally distributed. In addition, the normality of residuals was inspected to ensure the appropriateness of parametric analyses.

Accordingly, non-parametric methods were applied to EEG data, while parametric analyses were used for behavioral outcomes. To accommodate non-normal data within a factorial design, EEG data were analyzed using the Aligned Rank Transform (ART), which enables non-parametric analysis of variance in factorial designs. Specifically, a three-way mixed-design ANOVA based on ART was conducted, with group (mindfulness vs. control) as a between-subject factor, and region of interest (ROI: frontal, central, parietal, temporal, and occipital) and training phase (pre-test, post-test, follow-up) as within-subject factors. These analyses were performed separately for each frequency band (delta, theta, alpha, beta, and gamma).

For shooting performance, a 2 (group) × 3 (training phase) mixed-design ANOVA was conducted. For mindfulness scores, a repeated-measures ANOVA was performed. Effect sizes were reported using partial eta squared (η*p*^2^).

When significant main effects or interactions were observed, *post-hoc* pairwise comparisons were conducted. All *post-hoc* comparisons and pairwise tests were adjusted for false discovery rate using the Benjamini–Hochberg procedure. Reported *q*-values represent the FDR-corrected significance level, with *q* < 0.05 considered statistically significant.

To further examine training-related changes, differences in power spectral density (PSD) between pre-test and post-test/follow-up were calculated. Associations between changes in behavioral measures (i.e., mindfulness scores and shooting performance) and PSD changes in specific brain regions were assessed using Spearman correlation coefficients (*r*_*s*_). Statistical significance of the correlations was determined using permutation testing (1,000 iterations) combined with FDR correction across all correlation tests.

## Results

### Manipulation check

To verify the effectiveness of the pressure induction procedure, athletes' anxiety scores were compared before and after the manipulation. Post-induction anxiety scores (*M* = 66.1, *SD* = 4.8) were significantly higher than pre-induction scores (*M* = 53.7, *SD* = 3.0), *t*(13) = −9.53, *p* < 0.001. These results confirm that the stress induction successfully created a competitive pressure state suitable for subsequent testing.

### Shooting performance of mindfulness intervention

Prior to the main analysis, between-group comparability at baseline was confirmed: the two groups showed comparable shooting performance at pre-test [A1; mindfulness group: *M* = 624.5, *SD* = 2.1; control group: *M* = 624.2, *SD* = 3.7; *t*(12) = 0.187, *p* = 0.855], indicating baseline equivalence (see Table 2).

**Table 2:** Shooting performance across all time points.

Time point-	Mindfulness group (*M* ±*SD*)	Control group (*M* ±*SD*)	*t*(12)	*p*-value
Pre-test (A1)	624.5 ± 2.1	624.2 ± 3.7	0.187	0.855
Post-test (A2)	628.0 ± 1.8	622.1 ± 2.9	4.573	<0.001
Follow-up (A3)	626.9 ± 1.9	624.4 ± 2.7	2.003	0.068

M, mean; SD, standard deviation.

A 2 × 3 mixed-design ANOVA showed a significant main effect of group, *F*_(1, 12)_ = 7.333, *p* = 0.019, η*p*^2^ = 0.379. The main effect of time, *F*_(2, 24)_ = 0.367, *p* = 0.695, η*p*^2^= 0.030, and the time × group interaction, *F*_(2, 24)_ = 1.907, *p* = 0.163, η*p*^2^ = 0.137, were not significant. Thus, differential change over time between groups was not statistically confirmed at the overall model level.

Given the small sample size (*n* = 7 per group), between-group differences at individual post-baseline time points were further characterized descriptively alongside inferential comparisons. Regarding shooting performance, the mindfulness group showed a descriptive within-group trend of improvement from pre-test (*M* = 624.5, *SD* = 2.1) to post-test (*M* = 628.0, *SD* = 1.8). Conversely, the control group showed no such trend, with scores changing from pre-test (*M* = 624.2, *SD* = 3.7) to post-test (*M* = 622.1, *SD* = 2.9). An exploratory between-group comparison at post-test (A2) indicated that the mindfulness group had a statistically higher mean score than the control group [*t*(12) = 4.573, *p* < 0.001, *q* < 0.001]; however, because the overall group × time interaction was not significant, this isolated difference cannot be interpreted as a valid differential intervention effect, and therefore should be viewed with extreme caution. Furthermore, at the 2-week follow-up (A3), the mean shooting score for the mindfulness group was *M* = 626.9 (*SD* = 1.9), and the control group scored *M* = 624.4 (*SD* = 2.7), with no statistically significant difference observed between the groups [*t*(12) = 2.003, *p* = 0.059, *q* = 0.068].

### Mindfulness level changes

The Shapiro–Wilk test indicated that all mindfulness variables met the normality assumption. Repeated-measures ANOVA revealed a significant main effect of time, *F*_(2, 24)_ = 45.27, *p* < 0.001, η*p*^2^ = 0.790. Mindfulness scores increased significantly from baseline (A1: *M* = 112.5, *SD* = 8.4) to post-test (A2: *M* = 145.3, *SD* = 7.9; *p* < 0.001, *q* < 0.001) and remained above baseline at follow-up (A3: *M* = 138.7, *SD* = 8.1; *p* = 0.006, *q* = 0.009).

A significant time × group interaction was detected, *F*_(2, 24)_ = 12.63, *p* < 0.001, η*p*^2^ = 0.513. Simple effects indicated that the mindfulness group improved significantly more than the control group at post-test (*p* < 0.001, *q* < 0.001) and follow-up (*p* = 0.003, *q* = 0.004). Group differences were significant at A2, *F*_(1, 12)_ = 34.18, *p* < 0.001, η*p*^2^ = 0.741, and at A3, *F*_(1, 12)_ = 27.46, *p* = 0.002, η*p*^2^ = 0.696, but not at baseline (*p* = 0.358, *q* = 0.377). These findings demonstrate that the mindfulness intervention effectively enhanced and partially sustained athletes' mindfulness levels.

### EEG results

Prior to the main analyses, independent *t*-tests confirmed no significant differences between groups at pre-test (A1) across all EEG frequency bands (all *ps* > 0.05), indicating baseline equivalence.

#### Delta band (1–4 Hz)

**Figure 2:**
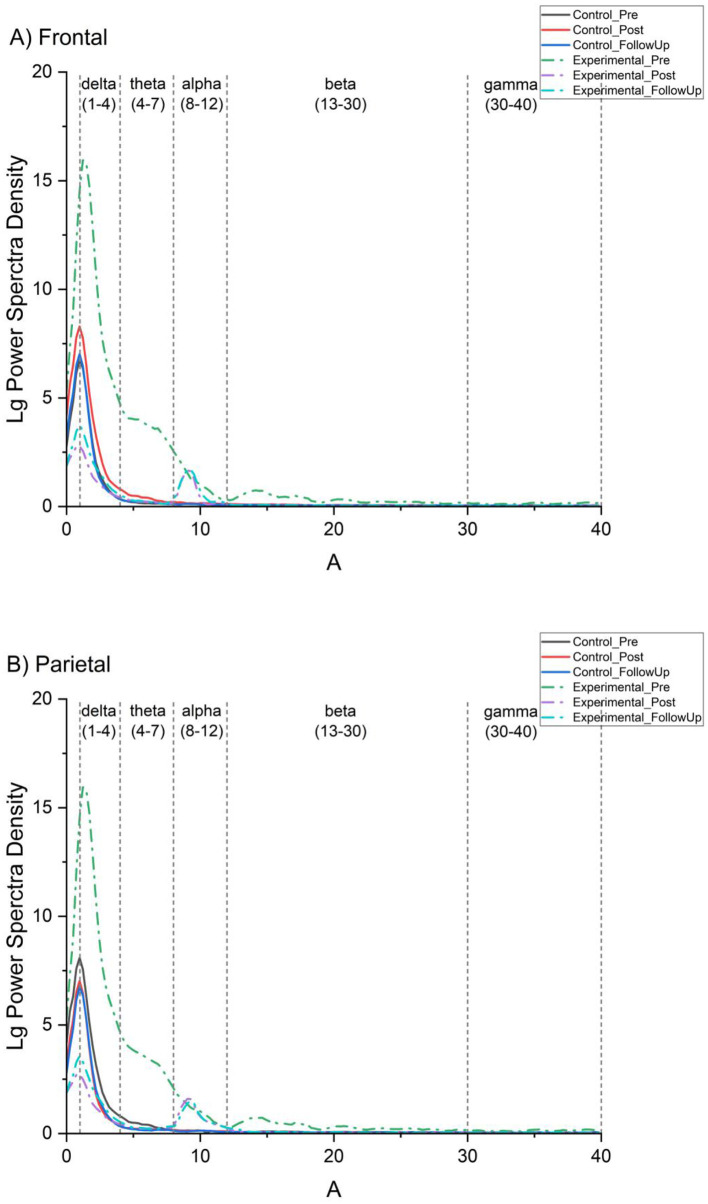
Grand-averaged power spectral density (PSD) of EEG signals across frequency bands in the frontal **(A)** and parietal **(B)** regions. Notes PSD values were averaged across electrodes within each region and are plotted as a function of frequency (0–40 Hz). Curves represent different experimental conditions for the control group (C) and training group (T) at three time points: pre-test (A1), post-test (A2), and follow-up (A3). Shaded frequency ranges and vertical dashed lines indicate canonical EEG frequency bands, including delta (1–4 Hz), theta (4–8 Hz), alpha (8–12 Hz), low beta (12–21 Hz), high beta (21–30 Hz), and low gamma (30–40 Hz). The figure illustrates the spectral distribution of neural activity and its modulation across groups and time points.

The three-way ANOVA revealed significant main effects of brain region, *F*_(4, 48)_ = 136.16, *p* < 0.001, η*p*^2^ = 0.919, and training phase, *F*_(2, 24)_ = 5.35, *p* = 0.012, η*p*^2^ = 0.308. The main effect of group was not significant, *F*_(1, 12)_ = 0.94, *p* = 0.351, η*p*^2^ = 0.073. None of the two-way or three-way interactions reached significance (*ps* > 0.05), indicating that the reduction in delta-band power over time was a shared pattern across both groups rather than a differential intervention effect. Follow-up comparisons on the significant main effect of training phase indicated significant reductions in delta-band power at post-test (*p* < 0.001, *q* < 0.001) and follow-up (*p* < 0.001, *q* < 0.001) relative to pre-test, collapsed across groups (Figure 2).

#### Alpha band (8–12 Hz)

The three-way ANOVA revealed significant main effects of training phase, *F*_(2, 24)_ = 43.91, *p* < 0.001, η*p*^2^ = 0.785, and group, *F*_(1, 12)_ = 7.78, *p* = 0.017, η*p*^2^ = 0.393. The main effect of brain region was not significant, *F*_(4, 48)_ = 1.03, *p* = 0.334, η*p*^2^ = 0.079. A significant training phase × group interaction was observed, *F*_(2, 24)_ = 5.62, *p* = 0.028, η*p*^2^ = 0.319 (Figure 2). No other interactions were significant (*ps* > 0.05). Follow-up comparisons indicated that alpha-band power increased significantly within the mindfulness group from pre-test to post-test (*p* < 0.001, *q* < 0.001) and to follow-up (*p* = 0.019, *q* = 0.023), whereas no significant within-group changes were observed in the control group (*ps* > 0.05, *qs* > 0.05).

Given the small sample size (*n* = 7 per group), between-group differences at individual post-baseline time points were characterized descriptively. At post-test (A2), alpha levels remained comparable between groups (mindfulness group: *M* = 0.890, *SD* = 0.177; control group: *M* = 0.852, *SD* = 0.291). A similar pattern was observed at follow-up (A3; mindfulness group: *M* = 0.737, *SD* = 0.152; control group: *M* = 0.793, *SD* = 0.245). These descriptive patterns suggest that the significant interaction reflects differential within-group trajectories over time, rather than a large absolute between-group difference at any single time point.

#### Theta band (4–8 Hz)

No significant main effects were observed for training phase, *F*_(2, 24)_ = 2.61, *p* = 0.094, η*p*^2^ = 0.179, group, *F*_(1, 12)_ = 0.72, *p* = 0.413, η*p*^2^ = 0.057, and brain region, *F*_(4, 48)_ = 0.65, *p* = 0.438, η*p*^2^ = 0.051. In addition, no significant two-way or three-way interactions were detected (*ps* > 0.05). Follow-up comparisons indicated no significant differences between any time points (*ps* > 0.05, *qs* > 0.05).

#### Beta bands (12–30 Hz)

No significant main effects were observed for training phase, *F*_(2, 24)_ = 0.79, *p* = 0.429, η*p*^2^ = 0.062, group, *F*_(1, 12)_ = 0.89, *p* = 0.376, η*p*^2^ = 0.069 or brain region, *F*_(4, 48)_ = 1.05, *p* = 0.327, η*p*^2^ = 0.080. Likewise, none of the two-way interactions were significant, and the three-way interaction was also not significant (*ps* > 0.05).

#### Gamma bands (30–40 Hz)

A significant main effect of brain region was observed, *F*_(4, 48)_ = 104.13, *p* < 0.001, η*p*^2^ = 0.897, indicating marked regional differences in gamma-band power. No significant main effects were found for group, *F*_(1, 12)_ = 0.92, *p* = 0.358, η*p*^2^ = 0.071, or training phase, *F*_(2, 24)_ = 1.24, *p* = 0.293, η*p*^2^ = 0.094. No significant two-way or three-way interactions were observed (*ps* > 0.05), indicating no differential intervention effect on gamma-band power across groups or time points.

### Correlation analysis between EEG results and behavioral measures

To further disentangle the specific neurocognitive mechanisms underlying the intervention, an exploratory analysis was conducted. Acknowledging the lack of significant interaction effects in the behavioral and certain EEG data, we cautiously analyzed the associations between region-specific oscillatory dynamics (frontal and parietal) and behavioral outcomes in the experimental group.

#### Associations between mindfulness and neural modulation

Regarding the relationship between neural plasticity and the cultivation of mindfulness traits, the strongest coupling was observed in the frontal region. Specifically, a significant positive correlation was found between the increase in dispositional mindfulness and Frontal Alpha enhancement for short-term effects (*r*_*s*_ = 0.782, *p* = 0.038, *q* = 0.048). This association persisted as a marginally significant trend for long-term effects (*r*_*s*_ = 0.745, *p* = 0.055, *q* = 0.059). Additionally, the suppression of Frontal Delta power showed a marginally significant negative correlation with mindfulness improvements in the short term (*r*_*s*_ = −0.715, *p* = 0.071, *q* = 0.085) and maintained a similar trend in the long term (*r*_*s*_ = −0.680, *p* = 0.093, *q* = 0.094). In contrast, no significant correlations were found between parietal oscillatory dynamics and mindfulness changes. For Parietal Alpha power, correlations were non-significant for both short-term (*r*_*s*_= 0.415, *p* = 0.355, *q* = 0.363) and long-term effects (*r*_*s*_ = 0.390, *p* = 0.387, *q* = 0.402). Similarly, Parietal Delta suppression did not significantly correlate with mindfulness scores in either the short term (*r*_*s*_ = −0.280, *p* = 0.531, *q* = 0.548) or long term (*r*_*s*_ = −0.255, *p* = 0.581, *q* = 0.600).

#### Neural predictors of shooting performance

In terms of shooting performance, the correlation pattern shifted toward the parietal region. Oscillatory changes in the frontal region did not significantly predict performance gains. For Frontal Alpha power, correlations with shooting improvement were non-significant for both short-term (*r*_*s*_ = 0.512, *p* = 0.240, *q* = 0.251) and long-term effects (*r*_*s*_ = 0.535, *p* = 0.215, *q* = 0.228). Likewise, Frontal Delta suppression showed no significant association with performance in the short term (*r*_*s*_ = −0.485, *p* = 0.270, *q* = 0.275) or long term (*r*_*s*_ = −0.510, *p* = 0.242, *q* = 0.253). However, a distinct pattern emerged in the parietal region. For short-term effects, Parietal Alpha enhancement showed a positive correlation trend with shooting performance improvement (*r*_*s*_ = 0.655, *p* = 0.110, *q* = 0.126). Notably, for long-term retention, this correlation strength increased to a marginally significant level (*r*_*s*_ = 0.725, *p* = 0.065, *q* = 0.077). However, Parietal Delta suppression did not significantly correlate with performance gains in either the short term (*r*_*s*_ = −0.350, *p* = 0.425, *q* = 0.442) or long term (*r*_*s*_ = −0.385, *p* = 0.394, *q* = 0.408).

## Discussion

This preliminary study investigated the potential effects of a 7-week MAIC-based mindfulness training programme on psychological responses, neural oscillatory activity, and shooting performance in elite air rifle athletes. Across multiple indicators, the intervention produced a coherent pattern of improvements, suggesting that MT may enhance attentional regulation and contribute to more efficient performance under competitive stress. However, it is important to acknowledge at the outset that the group-by-time interactions for some key measures, notably shooting performance, did not reach statistical significance. Therefore, the observed benefits primarily reflect within-group progress rather than definitive statistical superiority over the control group, and the subsequent discussions of these intervention effects should be interpreted with caution.

### Mindfulness may enhance athletes' shooting performance under pressure

Regarding behavioral findings, although the group × time interaction was not significant (indicating performance was not significantly improved compared to the control group), a 7-week MAIC mindfulness training programme was associated with a pattern of within-group improvement in the shooting performance of elite rifle athletes under competitive stress, and this positive effect was maintained at the 2-week follow-up test post-intervention. This result aligns with phenomena previously observed in other precision sports, confirming the robustness of mindfulness as an effective performance enhancement strategy. For instance, this consistency is echoed in the work of Ding et al. (2025), who observed that long-term mindfulness training significantly improved attention stability and reduced physiological stress markers (such as cortisol) in professional fencers, another discipline requiring acute focus. Similarly, a study by Wu et al. (2021) found that a 4-week mindfulness-based peak performance intervention significantly improved the shooting scores of archers. This indicates that the benefits of mindfulness can extend across different precision sport disciplines. Furthermore, the findings of the present study bridge previous discoveries across athlete populations of varying skill levels. Research by Guo et al. (2025) demonstrated that even a brief 18-day mindfulness meditation intervention effectively enhanced the shooting performance of novice athletes. This effect appears to be translatable across motor domains, as Wagner and Wieczorek (2024) demonstrated that brief mindfulness interventions could successfully counteract the detrimental effects of ego-depletion on motor skills in basketball and football tasks under pressure. By confirming that these positive effects are also present in elite athletes, the current study supports the view that mindfulness serves as a universal resource for motor preservation. More importantly, the link between mindfulness and performance is not confined to intervention efficacy but is also manifested in athletes' inherent traits. In a prospective study of elite biathletes, Josefsson et al. (2021) found that athletes' trait mindfulness levels were effective predictors of their shooting performance in actual competition. This trait-level benefit is further substantiated by Lee et al. (2024), whose research on young judo athletes highlighted that mindfulness practice actively buffers against depression and anxiety while enhancing resilience, thereby preserving the mental resources necessary for competition.

The findings of the present study build upon and extend the conclusions of previous research. Prior long-term intervention studies, such as the 7-week mindfulness acceptance commitment programme for sub-elite squash players conducted by Wong et al. (2022) and the 6-week specialized training for rowers by Sparks and Ring (2022), have already confirmed that mindfulness can enhance subjective evaluations of athletic performance by coaches or athletes. While these studies validate the efficacy of long-term training, they primarily rely on subjective reports. As Hooi et al. (2025) noted in their heart rate variability study, while subjective stress reductions are commonly reported, distinguishing specific objective improvements in cognitive flexibility often requires more rigorous measurement than self-report alone. This raises the question of whether this effect can be substantiated through objective metrics in precision sports. The study by Gao and Zhang (2023) took a critical step in this direction within the domain of shooting (albeit in a virtual reality environment). Employing objective measures of fNIRS and eye-tracking, they convincingly demonstrated that a brief 20-min mindfulness meditation session was sufficient to improve the attentional control and motor performance of shooting novices. Such improvements in attentional control under stress are theoretically grounded in the neural framework described by Paulus (2016), which suggests that mindfulness interventions moderate the impact of stress by enhancing top-down cognitive control regions. However, whereas Gao and Zhang (2023) investigated the immediate effects of a brief intervention on novices—a finding that Teng et al. (2022) suggest may be linked to the brain's ability to maintain a “task-ready” functional connectivity state after stress exposure—the present study explores whether long-term, structured training can yield measurable, objective improvements in both neural mechanisms (potentially involving the enhanced network connectivity observed by Bremer et al., 2022) and performance within a population of elite shooting athletes.

### Alpha enhancement reflects improved top-down control

In cognitive neuroscience, the enhancement of alpha power is widely regarded as a key indicator of attentional control, a process deeply rooted in the top-down modulation of anticipatory rhythms by the posterior parietal cortex (Capotosto et al., 2008). Its core mechanism is understood as “functional inhibition”: the brain actively utilizes alpha oscillations to suppress or reduce activity in brain regions processing task-irrelevant information (Bonnefond and Jensen, 2025). Recent evidence suggests that this active suppression not only blocks distractors but also sharpens the temporal precision of attentional sampling, thereby accelerating visual awareness (Yang, 2025). The core finding of the present study is the distinct pattern of alpha power enhancement observed within the mindfulness group, which was accompanied by an improvement in shooting performance. Although the absolute between-group differences at post-test did not reach statistical significance, this longitudinal within-group enhancement remains highly consistent with this theory, underscoring the role of alpha oscillations in stabilizing attentional focus. This result mirrors the classic “psychomotor efficiency” profile observed in skilled marksmen, where increased alpha activity specifically reflects the inhibition of task-irrelevant left-hemispheric analytical processes to optimize visuospatial performance (Hatfield et al., 1984). This perspective is strongly supported by related causal research; for example, Clayton et al. (2019) found that artificially enhancing alpha oscillations via transcranial alternating current stimulation could prevent performance decline due to fatigue in participants during a sustained visual attention task. In other words, alpha oscillations act as a “performance stabilizer,” fostering a state of “relaxed alertness” that allows athletes to maintain focus without excessive neural expenditure (Lomas et al., 2015). This ability to maintain efficient cognitive regulation under high pressure is, in itself, a manifestation of advanced cognitive function. In fact, the baseline efficiency of the alpha system has been shown to positively correlate with superior cognitive performance (Rathee et al., 2020). Crucially, such efficiency is not static; short-term training can fundamentally alter resting-state alpha dynamics, creating a more efficient neural substrate for subsequent performance (Rusinova et al., 2024; Klimesch, 1999). The achievement of this cognitive efficiency depends on the fine-grained control of attentional resources by alpha oscillations: when an individual performs a high-load primary task, the brain must actively inhibit irrelevant sensory channels. Callan et al. (2023) clearly demonstrated this in a study on “attentional deafness,” finding that when individuals “missed” auditory signals while performing a visual-motor task, this precisely corresponded to a significant enhancement of alpha power in their auditory cortex. This finding aligns with the global dynamics of selective attention, where alpha-dominated states in sensory cortices serve to gate external inputs to protect internal processing resources (Lakatos et al., 2016). In summary, we posit that the alpha power enhancement observed in our study is a neural marker of enhanced top-down attentional control in athletes. This enables them to more effectively suppress internal (e.g., anxiety, stray thoughts) and external distractions, thereby achieving more stable competitive performance. While the RCT design excludes external confounds, the exploratory correlation analysis provides preliminary support for the intervention's mechanism. The association between Frontal Alpha enhancement and dispositional mindfulness provides preliminary support for the notion that these benefits may derive from specific neural adaptations reflecting “internal silencing,” rather than non-specific placebo effects. However, given that between-group comparisons at post-test did not reach statistical significance, these interpretations should be regarded as exploratory.

### General reduction in delta power may reflect shared neural adaptations to competitive stress

The reduction in Delta band power, observed across both the mindfulness and control groups over the testing phases, suggests a state of reduced cognitive fatigue and optimized neural efficiency. This aligns with Schroeder and Lakatos (2009), who propose that the brain shifts to a “vigilance mode” by suppressing low-frequency oscillations to maximize sensitivity. While Ramirez-Moreno et al. (2021) linked Delta to low arousal, our findings extend this specifically to the athletic context: as elite marksmen enter the highly focused “shooting state,” the brain actively suppresses sensory-motor Delta rhythms, preventing “idling” states during performance execution (Tei et al., 2009).

Furthermore, Delta wave activity has been identified as a longitudinal marker of fatigue (Dybvik et al., 2025), potentially reflecting the re-emergence of ancient homeostatic drives when cortical control fails (Knyazev, 2012). Therefore, the overarching Delta power reduction observed in our study suggests that the athletes' rigorous routine training and their progressive habituation to the competitive stress protocol may have helped mitigate this fatigue accumulation over time. Mechanistically, since Delta amplitude linearly correlates with cerebral metabolic rate (Alper et al., 2006; Boord et al., 2007), this reduction signifies a shift away from the “high metabolic-cost activation” typically seen under acute stress (Kadziolka et al., 2016). Ultimately, as athletes became more adapted to the competitive environment, both groups were able to allocate cognitive resources more efficiently during the shooting task.

### Theta trend: potential markers of early cognitive regulation

In the present study, we observed a trend of increased theta band power post-intervention, a physiological pattern identified by Lomas et al. (2015) as a robust signature of mindfulness-induced “relaxed alertness” and emotional regulation, although this did not reach statistical significance. Crucially, because no significant interaction effects were found for the theta band, the interpretation of these mindfulness-related neural changes must be made with caution. While this trend broadly aligns with the hypothesis that MT is intended to enhance executive control functions. Indeed, theta activity—particularly in the prefrontal cortex—has been identified as a key marker of training-induced neuroplasticity. For example, research by Zhang et al. (2025) explicitly found that successful actual motor training significantly induces an increase in prefrontal cortex theta power, and that this change is significantly correlated with enhancements in sensory discrimination. However, the lack of statistical significance suggests the modulation is subtle. As Lee et al. (2018) argue, theta dynamics are not monolithic but index specific internalized monitoring mechanisms that vary dynamically by meditative stage. This point is corroborated by the findings of Bailey et al. (2020), who found that experienced meditators differed from controls not in overall theta power, but in oscillation stability. Consequently, the benefits of MT may reflect neural efficiency rather than mere activity strength. This distinction is critical when considering Wascher et al. (2014), who demonstrated that substantial increases in frontal theta often track accumulating mental fatigue and compensatory cognitive effort. Thus, the absence of a drastic theta spike in our data may conversely signal an optimized neural efficiency—achieving cognitive control without the metabolic cost of compensatory exhaustion—consistent with the network reorganization observed in various training modalities.

### No changes in beta/gamma: specificity of mindfulness effects

The absence of significant changes in beta and gamma oscillations suggests that the MAIC-based mindfulness intervention exerted a selective influence on neural dynamics associated with attentional regulation, rather than inducing broad, unspecific alterations in cortical activity. Contemporary models conceptualize beta activity not as a sustained rhythm, but as transient high-amplitude bursts supporting intermittent functional inhibition during goal-directed control (Lundqvist et al., 2024). The stability of beta power observed here indicates that mindfulness training enhanced attentional stability without fundamentally modifying these burst-dependent inhibitory mechanisms. Similarly, gamma activity is widely considered a marker of local cortical processing, particularly within sensorimotor regions, where it supports fine-grained information integration (Frauscher et al., 2018). Although high-frequency gamma synchronization has been linked to large-scale neural integration and feature binding (Uhhaas and Singer, 2006), the lack of gamma modulation in the present study suggests that mindfulness training did not restructure these fast, local processing architectures. Instead, the results are consistent with a model in which mindfulness selectively refines top-down attentional gating, while leaving high-frequency synchronization mechanisms supporting basic sensorimotor processing intact.

## Limitations

Despite its contributions, this study has several limitations that should be addressed in future research. First and most notably, as a methodological limitation, the extremely small sample size (*n* = 14) significantly reduces statistical power, which raises valid concerns for an EEG-based intervention study. Although the relatively small sample size may limit the generalizability of the findings and increase the risk of Type II errors, it is important to note that recruiting elite-level athletes—especially those competing in precision sports at the provincial or national level—poses substantial practical challenges (Yarrow et al., 2009). In addition, eliciting measurable performance improvements in such a highly trained population is inherently difficult, as their skills are already near ceiling (Pan et al., 2026). Consequently, regarding shooting performance, while descriptive patterns and *post-hoc* comparisons indicated within-group improvements and a nominally significant between-group difference at post-test, these findings lack robust statistical support from the overall interaction effect and must be interpreted with caution. Therefore, despite the limited number of participants and the resulting constraints on statistical power, the observed neurobehavioral enhancements following mindfulness training hold notable theoretical and practical significance, underscoring the potential of structured psychological interventions even among elite performers. Regarding the neurophysiological measures, although EEG provided valuable temporal insights into neural oscillatory dynamics, its limited spatial resolution precluded precise localization of cortical areas involved in attentional modulation. Moreover, while a significant group × time interaction was observed for the alpha band, no significant interaction effects were found in the delta, theta, beta, or gamma bands. Consequently, interpretations regarding mindfulness-specific neural changes in these non-alpha frequency bands should be made with caution, and our exploratory correlational findings must be viewed as preliminary. The relatively short duration of the mindfulness intervention also constrains conclusions about the persistence of training effects over time. Additionally, although the inclusion of a control group helped mitigate confounding factors, expectancy or placebo effects could not be entirely ruled out. Another methodological limitation exists regarding the administration of the CSAI-2 questionnaire. Although a deliberate temporal interval was incorporated between the initial baseline assessment and the stress induction procedure, participants ultimately completed the inventory twice on the same day. While this repeated measurement is acceptable and necessary as a manipulation check to verify the efficacy of the stress protocol, such frequent exposure within a short timeframe may introduce testing effects, potentially leading to habituation or influencing the athletes' subjective reporting of their anxiety states. Finally, due to the limited statistical power and the absence of significant interaction effects in both the behavioral and several EEG measures, the exploratory correlation results should be interpreted with strict caution. The focused ROI analysis on the frontoparietal network, while theoretically justified for attentional regulation, may have also overlooked intervention effects in other relevant cortical areas. Given these methodological constraints, future research should therefore employ larger samples and longitudinal designs to adequately power the statistical models, examine the long-term stability of mindfulness-induced benefits, and integrate multimodal neuroimaging methods and EEG source localization techniques (Wang et al., 2021, 2022; Yao et al., 2024) alongside additional analytical approaches such as EEG coherence to provide more fine-grained spatial information about cortical and subcortical mechanisms (Wang et al., 2023). Moreover, exploring individual difference variables—such as attentional style, or anxiety sensitivity—may help clarify the boundary conditions of mindfulness effectiveness. Extending this research framework to other precision or endurance sports would further validate the generalizability and robustness of the mindfulness–attention–performance pathway.

## Conclusions

This preliminary study suggests that a 7-week MAIC mindfulness training programme may enhance attentional regulation and is associated with within-group trends in shooting performance in elite rifle athletes under competitive stress. Confirmatory EEG results show increased alpha power associated with the intervention, alongside a generalized decrease in delta power reflecting shared task habituation. Collectively, these changes indicate improved neural efficiency, which in exploratory analyses correlates with better shooting accuracy. Behavioral improvements were maintained at follow-up. These findings provide preliminary neurobehavioral evidence supporting mindfulness as a potentially effective, sustainable intervention for precision sports. Future research should expand sample sizes and employ multimodal neuroimaging to examine long-term effects and individual mechanisms.

## Data Availability

The raw data supporting the conclusions of this article will be made available by the authors, without undue reservation.
